# Responses of soil organic carbon cycle to land degradation by isotopically tracing in a typical karst area, southwest China

**DOI:** 10.7717/peerj.15249

**Published:** 2023-05-15

**Authors:** Ruiyin Han, Qian Zhang, Zhifang Xu

**Affiliations:** 1 Institute of Geology and Geophysics, Chinese Academy of Sciences, Beijng, China; 2 University of Chinese Academy of Sciences, Beijing, China; 3 Institute of Geographic Sciences and Natural Resources Research, Chinese Academy of Sciences, Beijing, China; 4 CAS Center for Excellence in Life and Paleoenvironment, Beijing, China

**Keywords:** Soil organic carbon, Stable carbon isotope, Land management, Karst soils, Isotopic tracing

## Abstract

**Background:**

The loss of soil organic carbon (SOC) under land degradation threatens crop production and reduces soil fertility and stability, which is more reflected in eco-sensitive environments. However, fewer studies simultaneously compared SOC variations and *δ*^13^C_SOC_ compositions under diverse land uses, especially in karst areas.

**Methods:**

Soil profiles from two agricultural lands and a secondary forest land were selected to analyze SOC contents and their stable isotope composition (*δ*^13^C_SOC_) in a typical karst area located in southwest China to understand the response of the SOC cycle to land degradation. Moreover, the relationships between SOC contents and mean weight diameter (MWD) and soil erodibility (K) factor were comprehensively analyzed for assessing the response of SOC to soil degradation risk.

**Results:**

The mean SOC content was found to be the lowest in abandoned cropland (6.91 g/kg), followed by secondary forest land (9.31 g/kg) and grazing shrubland (34.80 g/kg), respectively. Meanwhile, the *δ*^13^C_SOC_ values exhibited the following trend: secondary forest land (mean: −23.79‰) ≈abandoned cropland (mean: −23.76‰) >shrubland (mean: −25.33‰). The isotopic tracing results suggested that plant litter was the main contributor to SOC in the secondary forest land. Whereas abundant nitrogen from goat feces enhanced plant productivity and resulted in additional accumulation of SOC in the grazing shrubland. Conversely, long-term cultivation led to the depletion of SOC sequestration by the loss of calcium. In surface soils, the fractionation of *δ*^13^C_SOC_ were considerably affected by the decomposition of SOC by soil microorganisms and covered vegetation rather than agricultural influences.

**Conclusions:**

The findings indicate that the cycling of SOC and soil stability in the calcareous soil of southwest China are largely regulated by different land uses and the presence of vegetation cover. The depletion of SOC and soil physical degradation pose significant challenges for abandoned cropland, particularly in the karst area, where land degradation is inevitable. Nevertheless, moderate grazing enhances SOC levels, which is beneficial to the land fertility maintenance in the karst region. Therefore, more emphasis should be placed on the cultivation methods and management strategies for abandoned cropland in the karst area.

## Introduction

Soil acts as a crucial pool with terrestrial carbon, whose organic carbon stock is much higher than the total stocks of vegetation and the atmosphere (Wang et al., 2022b). The release of carbon (CH_4_ or CO_2_) from the decomposition of soil organic matter (SOM) may severely influence the dynamic balance of carbon on a global scale (Lehmann & Kleber, 2015). Generally, the storage of soil organic carbon (SOC) represents the soil fertility in a region and is widely regarded as an important indicator for evaluating soil stability (Wang et al., 2022c). Understanding the sequestration and turnover of SOC is essential for the regional carbon cycle. The storage of SOC in different regions (504 Pg to 3000 Pg) is characterized by high spatial heterogeneity (Scharlemann et al., 2014), which may result from chemical disturbance (*e.g.*, microorganism decomposition, fertilizer inputs, and plants uptakes), soil texture, land uses, and self-structure (An et al., 2010; Chen et al., 2012; Liu, Han & Li, 2021). Sustained inputs of organic fertilizers over an extended period can effectively maintain soil fertility and promote high crop yields (Abrar et al., 2021; Han et al., 2020). Nevertheless, the decrease of SOC contents widely occurs in abandoned croplands (Chang et al., 2017; Li et al., 2017; Xiao et al., 2022), and the soil texture and SOC composition are frequently modified after grazing (Wang et al., 2022b). Previous studies have emphasized the sequestration and turnover rates of soil carbon under different land uses (Chen et al., 2002; Hu & Lan, 2020; Zhao et al., 2021). Further research is necessary to elucidate the underlying mechanisms that govern SOC stability and carbon migration in response to varying land-use patterns, particularly in the context of karst areas.

Stable isotope ratios are considered as efficient tracers of the sources and transformation process of individual elements in ecological systems (Jou et al., 2021; Li et al., 2022; Liu et al., 2023; Liu et al., 2018; Zeng et al., 2023). For instance, the variation of stable carbon isotope ratio (defined as *δ*^13^C_SOC_) is widely applied to trace the turnover or decomposition of SOC in terrestrial ecosystems (Chen et al., 2002; Jou et al., 2021; Wynn, 2007). Besides climate, the fractionation of *δ*^13^C_SOC_ generally occurs during SOC in the plant-soil-microorganism cycle (Philben et al., 2022). The plants prefer to assimilate the ^13^C-depleted carbon, which is more pronounced than the selection processes used by microorganisms for utilization (Billings & Richter, 2006). A lighter carbon isotope is also emitted through soil respiration (CO_2_ and CH_4_) at the beginning, and the residual carbon is consistently assimilated by soil organisms and eventually returns to the soil as secretions or carcasses (Wynn, 2007). The increase of *δ*^13^C_SOC_ values also may be attributed to the mineralization of carbon with the soil deposition time in deeper soil (Philben et al., 2022). But in recent years, human activities (such as tillage, grazing, and deforestation) have strongly disrupted the functioning of the soil ecosystems, thereby altering the distribution and isotopic composition of SOC in soils (Billings & Richter, 2006; Liu, Han & Zhang, 2020; Wang et al., 2022b). Extensive studies have described the distribution law of SOC contents and the variation of carbon isotopic composition over time in a suit soil profile (*e.g.*, forest land, cropland, and abandoned cropland) (Billings & Richter, 2006; Loss et al., 2017; Xiao et al., 2022). However, fewer studies compared SOC variations and *δ*^13^C_SOC_ compositions under diverse land uses simultaneously, especially in karst soils. The comprehensive effect of various cover types of vegetation and complex soil properties on the carbon cycle still needs further investigation. Soil carbon composition characteristics are essential indicators for soil stability and carbon cycle processes, the knowledge of which benefits the optimization of soil management and land-use planning.

The karst area locates in southwest China, covering an area of over 500,000 km^2^, represents the largest karst region globally and is deemed a potential carbon sequestration pool (Liang et al., 2016). However, the ecological rehabilitation in the karst area is confronted with considerable challenges due to the high sensitivity and ecological fragility, which hinder recovery to the original ecological state. Soil layers in the karst area are also relatively shallow to other soils and severely affected by stony desertification (Chang et al., 2018). Agricultural activities may greatly affect the distribution of soil properties and compositions (Li et al., 2017). However, agriculture constitutes the predominant industry in most areas of Guizhou Province, which may potentially lead to nutrient loss and soil erosion. Despite widespread reports examining the disturbance of soil composition during cultivation (Gupta Choudhury et al., 2014; He et al., 2015; Qu & Han, 2023), few discussions have been carried out to the subsequent effects though grazing and terminate farming. The discussion of SOC stability and composition is essential to evaluate the rationality of land utilization in the karst region. Tongren City, a typical karst area has the 5th highest level of SOC storage in Guizhou Province (Zhang et al., 2019). The SOC storage under diverse land was in the order: forest land >cropland >grasssland ≈ uncultivated land in soils over 100 cm of Tongren City (Zhang et al., 2019). The analysis of SOC distribution under diverse land uses in the karst area can prevent soil nutrient loss in advance. This study explored soil profiles under different land-use types and managements in Tongren City to: (1) identify the influencing factors of SOC storage, (2) clarify the mechanism of *δ*^13^C_SOC_ fractionation, and (3) evaluate the disturbance of agricultural activities on the carbon cycle and provide theoretical evidence for the optimization of soil utilization and development in karst regions.

## Material and Methods

### Study area

**Figure 1:**
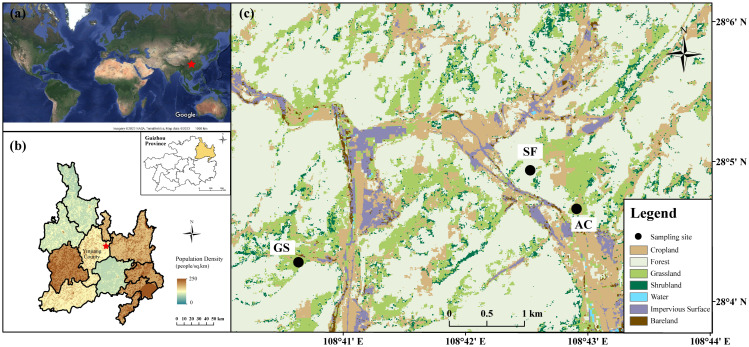
Sampling sites in Yinjiang County. (A) Map of the world (Map data^©^ 2022 Google), (B) Location and population density of Tongren city (Fu, Jiang & Huang, 2014a; Fu, Jiang & Huang, 2014b), and (C) Land uses in the study area.

The sampling area situates in Yinjiang county within the territory of Tongren city, the northeast of Guizhou Province (Fig. 1), which is characterized by karst landform with abundant mountains. Yinjiang county widely stretches across 700–800 m in elevation, with an elevation difference exceeding 2,100 m (Han & Xu, 2022). According to the statistical results from the Yinjiang government, the forest coverage in Yinjiang county reached 68.37%. Statistics from the bureau reported that the dominant economic industry in Yinjiang county was agriculture; the proportion of crops seeded reached 64,471 hectares and that of cereals covered 37,833 hectares in 2016. The soils are classified as calcareous soils by the United States Department of Agriculture (USDA) classification standard, which are mostly derived from carbonate rocks with sporadic coal seams and silicate rocks. The sub-tropical monsoon climate of Yinjiang county is notable for its significant seasonality and simultaneous variations in precipitation and temperature. Most rainfall occurs between April and October each year (>70%). The temperature varied from −2.2 °C to 38.6 °C, and the annual precipitation was 1281.4 mm in 2016.

### Field sampling

Large amounts of cropland have been abandoned or converted back to natural land resulting from the ‘Grain-for-Green’ program in China in the last 20 years. The native vegetation and abandoned cropland are vastly distributed in Yinjiang county, which proved useful for examining how agriculture affects soils. Three land-use soil profiles were chosen to be sampled in Sept. 2016 (Fig. 1). The sum of 20 samples was obtained in the profile of secondary forest land (SF), 16 samples were collected in the profile of abandoned cropland (AC), and 11 samples were collected in the profile of shrubland (GS). The description of sampling methods and soil profile characteristics have been reported by Han, Zhang & Xu (2023) and Han & Xu (2022). The sampling intervals were defined as 5 cm in shallow soils (above 20 cm), and as 10 cm in soil depths below 20 cm. The descriptions of sampling sites are provided in Table 1.

**Table 1:** Main features of the three profiles in Yinjiang County.

Profile	Altitude (m)	Land uses^a^	Main vegetation types
SF	828	Subtropical evergreen broad-leaved secondary forest	*Platycarya strobilacea Sieb.et Zucc .* (C_3_)*, Melia azedarach L.* (C_3_)*, Quercus fabri Hance* (C_3_)*, etc.*
AC	892	Sloping farmland (abandoned 3 years before sampling)	Corn (C_4_) and potatoes (C_3_)
GS	776	Native sloping shrub-grass land	*Pyracantha fortuneana* (C_3_), *Castanea mollissima* (C_3_), *Lindera communis* (C_3_)

**Notes.**

a Other information can be found in Han & Xu (2022).

### Soil composition measurements

After plant residues and stones were removed from air-dried soil samples, two groups were divided. Following the passing of the ground soils through a two mm mesh sieve, one group of samples was used for the analysis of soil chemical properties. The soil and bedrock samples were digested with HNO_3_–HF–HClO_4_, and the Ca contents were determined by ICP–OES (Optima 5300DV; Perkin Elmer, Waltham, MA, US). The suspension of soil and water (2:5) was employed to determine soil pH by a glass electrode with a precision of ±0.05 (Han et al., 2020). Soil particles (0.002 mm for clay, 0.053 mm for silt, and 0.250 mm for sand) were classified by the USDA Soil Taxonomy, and separated in wet-sieving by a laser particle size analyzer (Mastersizer 2000; Malvern Panalytical, Malvern, UK) (Van Bavel, 1950). Residual soils were ground for particle size less than 150 µm, then digested for 24 h with 0.5 mol/L HCl and repeatedly washed with pure water (18.2 M Ω cm) until neutrality was reached to eliminate the disturbance of carbonates (Liu, Han & Zhang, 2020). Subsequently, samples were dried at 55 °C to measure SOC and *δ*^13^C_SOC_. The SOC contents were obtained on an elemental analyzer (Vario TOC cube; Elementar, Langenselbold, Germany) with a precision of ±0.1%. In addition, the actual SOC contents were calibrated due to the loss of inorganic carbon:

\begin{eqnarray*}\mathrm{SOC}=SO{C}_{M}\times \frac{{M}_{1}}{{M}_{2}} \times 10 \end{eqnarray*}

where the SOC_M_ (%) represents the measured values of the soil organic carbon (g/kg), the M_1_ represents the mass of soils after removing carbonates, and M_2_ represents the mass of soils before removing carbonates.

### Isotopic measurements

The carbon isotopes were measured by a stable isotope mass spectrometer (Thermo, MAT-253, USA) with a precision better than 0.1‰. A duplicate measurement was performed on each sample to ensure accuracy. The *δ*^13^C_SOC_ (^13^C_SOC_/^12^C_SOC_) values were relative to Vienna Pee Dee Belemnite (V-PDB), which is expressed in the unit of‰:

\begin{eqnarray*}{\delta }^{13}{\mathrm{C}}_{\mathrm{ SOC}}= \frac{{\delta }^{13}{\mathrm{C}}_{\mathrm{M}}-{\delta }^{13}{\mathrm{C}}_{\mathrm{V }}}{{\delta }^{13}{\mathrm{C}}_{\mathrm{V }}} \times 1000 \end{eqnarray*}

where M means measured samples, and V means V-PDB standard. All measurements were accomplished in the Institute of Geographic Sciences and Natural Resources Research, CAS.

### Calculation and data processing

#### Mean weight diameter

Aiming to evaluate soil resistance to erosion, the calculation of mean weight diameter (MWD) based on soil particle proportions is employed. In general, higher MWD values indicate more stability and resistibility are reflected in soil structures, and the formula is shown as follows (Gupta Choudhury et al., 2014):

\begin{eqnarray*}\mathrm{MWD}=\sum _{\mathrm{i}=1}^{\mathrm{n}}{\mathrm{X}}_{\mathrm{ i}}\times {\mathrm{M}}_{\mathrm{i}} \end{eqnarray*}

where *i* represents the soil particle sizes that 1, 2, and 3 indicate clay-sized fraction, silt-sized fraction, and sand-sized fraction, respectively; X_*i*_ (mm) represents the mean diameters of soil particles; and the M_*i*_ (%) represents the mass proportion of soil particles.

### Soil erodibility factor

Soil erodibility denotes the resistance to erosion that is induced by external influence, which can be evaluated by the soil erodibility K factor (Kiani & Ghezelseflo, 2016). Depending on the physical and chemical properties of soils, soil erodibility presents regional characteristics within a different area. Based on soil properties (*e.g.*, soil structure, SOC contents, and soil texture), several experiential models were established (Favis-Mortlock & Boardman, 1995; Pal & Chakrabortty, 2019). The Erosion Productivity Impact Calculator (EPIC) model calculates K (t ha h(ha MJ/mm)) using soil texture and SOC contents, and equation (4) has been modified to accord for Chinese soils (Zhang et al., 2008):

$\mathrm{K}=0.5158 \left\{ 0.2+0.3\times \exp \left[ -0.0256{\mathrm{S}}_{\mathrm{a}} \left( 1- \frac{{\mathrm{S}}_{\mathrm{i}}}{100} \right) \right] \right\} \times { \left( \frac{{\mathrm{S}}_{\mathrm{i}}}{{C}_{l}} +{S}_{i} \right) }^{0.3}$

$\times \left[ 1- \frac{0.25\mathrm{C}}{{\mathrm{C}}_{\mathrm{SOC}}+\exp \left( 3.72-22.95{\mathrm{C}}_{\mathrm{SOC}} \right) } \right] \times \left[ 1- \frac{0.7{\mathrm{S}}_{\mathrm{A}}}{{\mathrm{S}}_{\mathrm{A}}+\exp \left( -5.51+22.9{\mathrm{S}}_{\mathrm{A}} \right) } \right] -0.01383$

where the S_a_, S_i_, C_l_ represents the percent of sand, silt, and clay in the total soil particles; S_A_ represents the values of 1-S_a_/100, and C_SOC_ represents the SOC contents (%). And the units of K factors were omitted for simplifying analyses. The higher values of K factors indicated stronger soil anti-erodibility.

### Data processing

The relationships among SOC, *δ*^13^C_SOC_ and related parameters were analyzed by linear regression analysis, with the determination of the coefficient R and *p*-values by IBM SPSS Statistics 25, USA. All graphs were created by ArcMap 10.8 (Esri, Redlands, CA, USA) and Origin 2017 (OriginLab, Northampton, MA, USA).

## Results

### Profile characteristics under different land uses

**Figure 2:**
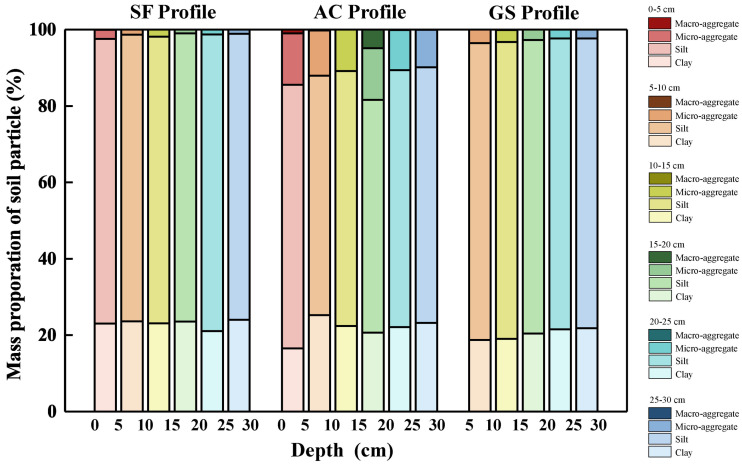
Soil particle distribution in the surface soil of the three profiles (0–30 cm).

Soil pH, particle distribution and MWD values are listed in Table S1. The detail of soil pH in three profiles was reported by Han, Zhang & Xu (2023): ranged from 7.1 to 7.9, 4.8 to 5.2, and 6.3 to 7.0 in the SF, AC, and GS profiles, respectively. The descriptions primarily focus on the soils above 30 cm) because the cycling of SOC is basically active in the surface soil. The distributions of soil particles in the surface soil (0 to 30 cm) of the three profiles are presented in Fig. 2. Obviously, silt occupied the largest proportion of soil particles among the three profiles. The sand in the AC profile occupied a larger proportion than that in others and decreased with increasing depth in the surface soil. In addition, the soil textures were uniform in the SF and GS profiles compared to those in the AC profile (Table S1). The mean values of MWD were in the order of that in the AC profile (2.14 mm) >GS profile (1.31 mm) >SF profile (1.24 mm). The values of MWD decreased above 100 cm and increased below 100 cm with depth in the AC profile, but little difference was observed between the SF and GS profiles.

### SOC content, *δ*^13^C_SOC_ value, and K factor

**Figure 3:**
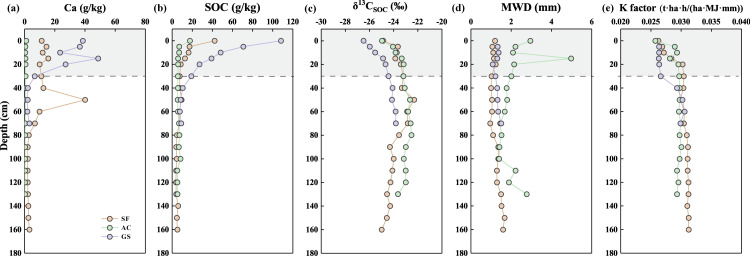
Vertical variation of (A) Ca contents, (B) SOC contents, (C) *δ*^13^*C*_SOC_ values, (D) MWD values, and (E) K factor in the three profiles.

The vertical distributions of SOC contents, *δ*^13^C_SOC_ values, MWD values, and K factor are illustrated in Fig. 3. The SOC contents ranged from 3.84 g/kg to 42.16 g/kg, 5.11 g/kg to 17.67 g/kg, and 7.63 g/kg to 108.19 g/kg in the SF, AC, and GS profiles, respectively. At the same soil depth of over 30 cm, the order of SOC and calcium (Ca) contents in the profiles was GS profile >SF profile >AC profile. A significant reduction in SOC content was observed with increasing depth below 30 cm in the SF and GS profiles, suggesting that human disturbance had a limited impact at greater depths. The values of *δ* ^13^C_SOC_ ranged from −25.00‰ to −22.30‰ in the SF profile, −24.97‰ to −22.52‰ in the AC profile, and −26.49‰ to −23.82‰ in the GS profile. High increasing rates of *δ*^13^C_SOC_ values were found in the 0 to 30 cm soil layers of three profiles. The values of *δ* ^13^C_SOC_ roughly showed an upward trend in the upper soils (above 50 cm) of the SF profile (rising by 2.55 ‰), while showing a downward (decreasing by 2.69‰) towards the bedrock (*δ*^13^C_SOC_ = −26.73‰) at deeper depths. In contrast, the *δ* ^13^C_SOC_ values consistently increased with increasing depth in the GS profile (an increment of 2.68 ‰). Apart from the topsoil, the variation of *δ* ^13^C_SOC_ values was slight with the range of 1.53‰ in the AC profile. The *δ*^13^C_SOC_ values in the GS profile were obviously lighter than those in the other profiles at the same depth. The *δ*^13^C_SOC_ values in the soils over 70 cm of AC profile were similar to those in the SF profile at the same depth. The values of K factor were in the range of 0.0262 to 0.0266, 0.0243 to 0.0259, and 0.0262 to 0.0264 in the SF, AC, and GS profiles, respectively. The K factors exhibited an increasing trend with depth in the SL profile and a slight increase up to 100 cm followed by a decrease in the AC profile. Notably, a significant variation in K factors was observed between the soils above 30 cm (0.0263 to 0.0266) and those below 30 cm (0.0293 to 0.0306) in the GS profile.

## Discussion

### Effects of land use on SOC contents

The dynamics of SOC are mainly controlled by covered vegetation, soil structure, and soil erosion, which may affect the input, decomposition, and loss of SOC, respectively (Han, Li & Tang, 2015; He et al., 2016). The impact of lithology on the spatial distribution of SOC contents within a region is expected to be limited, given the similarity in soil mechanical compositions (Doetterl et al., 2015). All soil profiles were selected at a small catchment with similar soil parent materials and climate. Therefore, the soil evolutions affected by land uses may be the key factor affecting SOC contents. The vertical variations of SOC and Ca in three profiles are presented in Fig. 3. Consistently, the contents of Ca followed the decreasing sequence of GS profile (mean values: 26.33 g/kg), SF profile (mean values: 13.05 g/kg), and AC profile (mean values: 0.47 g/kg). Generally, the stable formation of organic–inorganic complexes with Ca^2+^ in calcareous soil can strongly influence the stability of sequestrated SOC (Chen et al., 2012). The lower contents of Ca also may induce the loss of SOC. The possible explanation for the highest levels of SOC in the GS profile and the lowest levels in the AC profile, except for the SOC contents in 100 cm soil, may be attributed to the calcareous organic complexes.

Abundant microorganisms exist in the natural soils, especially in the surface soil, which can decompose SOC (Chen et al., 2002). The rapid decrease of SOC contents of the soil over 30 cm may result from the decomposition by abundant microorganisms (Han, Li & Tang, 2015). Plant residues provided continuous supplies of organic carbon to topsoil, and the SOC contents remained relatively constant due to the improvement of soil and water conservation by plant root (Sun, Zhu & Guo, 2015). The covered vegetation in the location of SF profile effectively improves the capacity of soil and water conservation to maintain SOC. Moreover, there was abundant plant residue to supply SOC to the soils in the SF profile. Although the SOC may be decomposed by soil microorganisms, some ions (*e.g.*, calcium and magnesium) can be complexed in a more stable form afterward (Heckman et al., 2009). The study area is characterized by carbonate rocks, where the natural soils have abundant calcium and magnesium to be conducive to fixing SOC (Li et al., 2017).

Except for the topsoil, the SOC contents were basically identical in the whole AC profile (range of 3.22 g/kg). The higher level of SOC in the topsoil may be attributed to the covered ruderal. After consuming SOC for growth, cultivated crops will never return to the soil at maturity. Furthermore, the decomposition rate of SOC is possibly improved by microbial biomass (Geisseler, Linquist & Lazicki, 2017). Additionally, the exposure time of soil will increase by frequent repeat tilling, and further consume SOC by decomposition and soil mineralization (Schjønning & Thomsen, 2013; Wang et al., 2022c). A mass of acidic compounds (*e.g.*, ammonium chloride and urea) will be added to the soil during the fertilizer application, which may decrease the soil pH (Čakmak et al., 2014). Ca and heavy metals are easily released into soil solutions in an acidic environment (Čakmak et al., 2014). Frequent irrigation can promote the loss of Ca, Mg, and heavy metals (Hall et al., 2001). Furthermore, the finer soil particles prefer to limit the external disturbance rather than accumulate the amount of SOC (Schjønning & Thomsen, 2013). Therefore, the SOC in the AC profile was difficult to maintain and store. The results indicated that nonnegligible SOC might be consumed in long-term cultivation, and difficulty recovering to the original level.

The location of the GS profile only experienced goats grazing in five years with no cultivated activities. Theoretically, the stock of C aboveground will decrease because the uptake of goats consumes part of returned SOC to the soil by the plant. However, SOC was concentrated in the surface soil (0–30 cm) of the GS profile. The SOC content was contained in the highest level of the topsoil (0–5 cm, 108.19 g/kg), which was 1.53 times greater than that in the 5 to 10 cm soil layer and 2.25 times greater than that in the 10 to 15 cm soil layer of the GS profile. Successive excretion of feces may increase biological productivity by abundantly supplying nitrogen, further increasing the uptake of CO_2_ by plants, and stimulating root biomass growth (Francisco et al., 2022; He et al., 2015). And the SOC inputs from plant debris may increase. Moreover, the formation of biogenic aggregates will be improved, which have higher sequestrated contents of nitrogen and carbon than physicogenic aggregates, especially in the surface soil (He et al., 2015). The Ca contents showed the highest level in the GS profile, which may be complexed with SOC in a stable formation. It is notable that the fastest decrease of SOC contents with depth was exhibited in the GS profile among the three profiles. There is also evidence that the effect of SOC contents in soils and roots from solid feces is mainly reflected in the topsoil (Francisco et al., 2022; Loss et al., 2017). The mean value of soil pH was 7.0 in the GS profile, in a neutral environment. In general, the Actinomycetes Strain is suitable for reproduction in a neutral environment, further improving the decomposition of SOC (Heckman et al., 2009). Moreover, the soil microorganisms can decompose SOC more effectively after acclimatizing to the combined environment of feces and soil (Wang et al., 2021). Accordingly, the SOC contents extremely decrease in the shallow soil layer of GS profile.

### Carbon cycle under diverse land uses by *δ*13C tracing

Previous studies found that vegetation type and moisture are the dominant factors of isotope fractionation during plants assimilation (Han, Li & Tang, 2015; Qu & Han, 2022; Yakir & Sternberg, 2000). The surficial debris contributes substantially to the carbon pool in soil (Yakir & Sternberg, 2000). Based on the pathways of carbon turnover during plant photosynthesis, the plants are mainly divided to C_3_ plants (use the Benson-Bassham-Calvin Cycle, −32‰ < *δ* ^13^C_SOC_ < −22‰) and C_4_ plants (use the Hatch-slack Cycle, −17‰ < *δ* ^13^C_SOC_ < −9‰) (Calvin & Benson, 1948; Johnson & Hatch, 1969; Kohn, 2010; Yakir & Sternberg, 2000). The C_3_ plants were the dominant vegetation in the study area, and the *δ*^13^C_SOC_ values in the three profiles ranged from −26.49‰ to −22.30‰. It can be inferred that carbon fractionation in the study area may be controlled by C_3_ plants, and the long-term cultivation of potatoes (C_4_) may have slightly changed *δ*^13^C_SOC_ composition in the AC profile. Additionally, the distributions of *δ*^13^C_SOC_ also have diverse characteristics under various land uses in a region (Jou et al., 2021). The turnover rate of SOC and carbon isotope fractionation in the cropland are lower than those in the natural land with limited human activities may be attributed to the lower amount of microorganisms and soil enzyme activity (Ni et al., 2015). The variation range of *δ*^13^C_SOC_ values was minimum in the AC profile (varied within 2.44‰), which was slightly smaller than that in the SF (varied within 2.69‰) and GS (varied within 2.68‰) profiles. The slight discrepancy indicates that the terminated cultivation may narrow the variation gap of *δ*^13^C_SOC_ values between cropland and natural land. Moreover, the soil parent material also influences the *δ*^13^C_SOC_ values in regional soils (Liu, Han & Zhang, 2020). The *δ*^13^C_SOC_ values were −26.73‰ and −28.77‰ in the bedrock of the SF and GS profiles, respectively. The proximity of SF and AC profiles, located at a distance less than 1 km, suggested a homogeneity in their geological origins. Therefore, it can be inferred that the *δ*^13^C_SOC_ value of the bedrock in the AC profile can be approximated by that in the SF profile. The lower *δ*^13^C_SOC_ values in the GS profile relative to other soil profiles may be inherited from bedrock.

**Figure 4:**
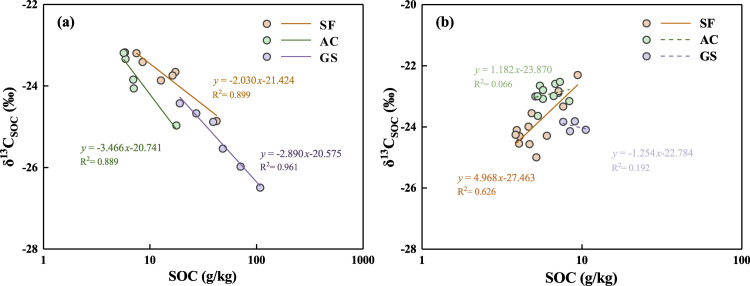
Correlation between SOC contents and *δ*^13^*C*_SOC_ values in the soils at 0 to 30 cm (A) and that in the soils below 30 cm (B) of the three profiles. The full line means *p* < 0.01, and the dashed line means *p* > 0.05.

The *δ*^13^C_SOC_ values are strongly affected by the decomposition of fresh SOM in the surface soil, while the old carbon in deeper soils is stable without evident isotopic fractionation (An et al., 2010). Therefore, the discussion between SOC contents and *δ*^13^C_SOC_ values is concentrated on soils from 0 to 30 cm in this study. The SOC contents and *δ*^13^C_SOC_ values had a negative correlation in the soils over 30 cm in three profiles (Fig. 4), and only had a positive correlation in the deep soil horizon of the SF profile. Although the utilization of CO_2_ during the respiration of soil organisms and plant roots may only trigger slight kinetic isotope fractionation, the selective decomposition of plant biochemicals can also affect the distribution of *δ*^13^C_SOC_ in soils (Breecker et al., 2015). Compared with the bulk SOC, the roots are generally enriched in ^13^C, whereas the ^13^C in roots may reduce in relation to that in leaves with plant growth (Philben et al., 2022). The decomposition of SOC with the enrichment of ^13^C cannot be entirely explained by roots decay in all profiles. In addition, it has been reported that the decrease in ^13^C levels was always accompanied by the increase of precipitation through reducing the decomposition rate of soil microorganism (Deng, Liu & Shangguan, 2014; Wang et al., 2022b). The soil microorganism activity may be restrained due to the decrease of the stomatal conductance of plants and dissolved oxygen under drought (annual precipitation lower than about 2000 mm) or excessively humid environments (annual precipitation higher than 3000 mm) (Xu et al., 2016). In contrast, a well-humid environment will increase SOC availability through the decomposition of plant debris and enrich ^13^C in soil by microorganism utilization (Deng, Liu & Shangguan, 2014; Peri et al., 2012). Since 1984, the annual precipitation has ranged from 800 mm to 1500 mm without extreme rainstorms or drought events in September (the sampling month) (Wu et al., 2017), which is suitable for the transport of SOC in the plant–soil–microorganism cycle to accumulate ^13^C. Moreover, ^13^C will continue to accumulate in the soil, along with the death of most soil microorganisms (Henn, Gleixner & Chapela, 2002). The additional input of soil organisms and fungi could make a substantial contribution to the enrichment of ^13^C, leading to the highest rate of increase in *δ* ^13^C_SOC_ (*δ*^13^C_SOC_ = 2.07‰) in the GS profile compared to the others, for depths over 30 cm. The accumulation of SOC accompanied by the enrichment of *δ*^13^C_SOC_ in deep soils of the SF profile may result from a mixing effect. The ^13^C dilution caused by bedrock weathering (*δ*^13^C_SOC_ value of −26.73‰) possibly prevents aboveground organisms from controlling SOC compositions.

### Assessment of soil degradation in the karst soil

**Figure 5:**
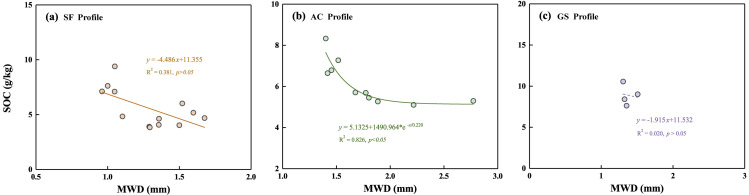
Correlation of MWD values and SOC contents in the soils below 30 cm of the three profiles. (A) SF profile, (B) AC profile, (C) GS profile.

The adverse influence of soil degradation on agriculture has severely restricted the development of the agriculture-driven economy in Guizhou Province. Obvious soil acidification had occurred in the AC profile (soil pH at an average of 5.0). The process of soil acidification may lead to excessive release of Ca, Mg, and heavy metals into soil solution, thereby reducing the storage of SOC and posing a threat to the growth of soil organisms and plants (Čakmak et al., 2014). Mn, Mo, Ni, Fe, and Cr exhibited reduced concentrations in the abandoned cropland compared to those in the uncultivated land as previously reported in Yinjiang county (Han & Xu, 2022). The above-mentioned analyses also demonstrated that agricultural practices significantly impacted the soil ecological system. Therefore, it is imperative to artificially protect cropland after abandoned rather than solely rely on the self-restoration of ecosystems. To better evaluate soil erosion, the soil aggregate stability index of MWD in different depths of three profiles was determined. SOC storage is more affected by soil aggregates rather than soil organisms in deeper soil (An et al., 2010). The MWD and SOC at deep soils (>30 cm) showed a negative linear correlation in the SF and GS profiles, and a negative curve correlation in the AC profile (Fig. 5). Generally, the majority of SOC and decayed plant residues are generally protected by larger soil particles, while part of SOC is immobilized in small soil particles (Adamczyk, Heinonsalo & Simon, 2020). Positive correlations between SOC contents and MWD values have been found in several areas (Gupta Choudhury et al., 2014; Li et al., 2015; Sun, Zhu & Guo, 2015), whereas the study soils showed the contrary. The soil particles were mainly composed of clay and silt (the mean proportions of 98.82%, 90.62%, and 97.34% in the SF, AC, and GS profiles, respectively), resulting in low MWD values in soils. Abundant fine-sized particles improve the sequestration of SOC by promoting the more stable formation of soil aggregates and mineral complexes (Huang & Hartemink, 2020). However, the soil texture will be coarsened in the short term after cropland abandonment because of the decreased capacity of cation exchange and SOC sequestration (Huang & Hartemink, 2020). Obviously, the loss of clay-silt particles was influenced by the cropland abandonment in the AC profile, which has adverse effects on protecting SOC sequestration. Conversely, moderate grazing levels may be conducive to protecting ecological balance and soil fertility. Moderate grazing even may compact the sandy soils to improve the cohesion forces of soils (Wang et al., 2022a).

Besides SOC contents and soil acidification, soil erodibility is also an essential indicator of soil degradation (He et al., 2016). The K factor was determined for evaluating soil erodibility in the three profiles. The range of K factor values (0.0259 to 0.0266) in the study area was in accordance with that of surface soils in Guizhou Province (0.0230 to 0.0477) but lower than that of most surface soils in southern China (0.0635 to 0.2183) (Zhang, De Angelis & Zhuang, 2011). It may be attributed to the high levels of SOC and silt proportion in Guizhou Province. The similar values of K factors in the SF and GS profiles at the same depth with a narrow range, suggest that moderate grazing did not affect soil erodibility. However, the K factors in the AC profile were inferior to those in other profiles, which may result from long-term cultivation on sloping farmland. The plant roots can strongly consolidate soil to improve soil texture, and the canopy of vegetation and litter layer can further reduce the splashing effect by precipitation (Sun et al., 2016). It is indicated that the soils in Yinjiang county are more susceptible to erosion, especially soils that have experienced cultivation. The management of slope farmland possibly increases soil erosion by rainfall erosivity, especially in karst areas (Li et al., 2015). The vertical distributions of SOC and K factor levels were dissimilar in the three profiles (*p* > 0.05). In general, an increase in sand apportion may reduce soil erodibility by increasing infiltration (Panagos et al., 2014). The lower levels of K factor and SOC on abandoned cropland should be noted. It is necessary to convert the existing form of traditional slope farming in Guizhou Province (such as the terrace). And the soil erodibility of terraces under reasonable management may also be greater than that of woodland (Jiang et al., 2020).

## Conclusions

The carbon cycling and soil stability were greatly controlled by different land uses in Yinjiang county. The agricultural practices significantly altered the soil texture and greatly enhanced the consumption of SOC. On the other hand, grazing activities could be the major contributor to the higher SOC contents in the shrubland than in other land uses, predominantly comprising ^13^C-depleted SOC. Basically, the *δ*^13^C_SOC_ fractionation ranges in 0 to 30 cm soils were limited by the C_3_ vegetation covered in the study area. The K factor values were at lower levels in surface soil and the values in abandoned cropland were lower than those in other profiles, which mainly controlled by soil particle distribution. The soil erosion risks are widely at a high level in the Guizhou Province due to the finer soils. Inadequate management practices, particularly under a monotonous cropping pattern, can substantially elevate the risk of soil erosion through decreased SOC accumulation and stability. Such practices can have significant long-term negative impacts. In order to promote the sustainable development of the eco-environment, it is essential to carefully plan the improvement of SOC composition and stability, as well as the restoration of abandoned cropland in karst regions.

##  Supplemental Information

10.7717/peerj.15249/supp-1 Supplemental Information 1 SOC contents and its isotope composition, and related parameters in the three soil profiles of the Yinjiang county

## Additional Information and Declarations

### Data Availability

The following information was supplied regarding data availability: The raw measurements are available in the Supplemental Files.

## References

[ref-1] Abrar MM, Xu H, Aziz T, Sun N, Mustafa A, Aslam MW, Shah SAA, Mehmood K, Zhou B, Ma X, Chen X, Xu M (2021). Carbon, nitrogen, and phosphorus stoichiometry mediate sensitivity of carbon stabilization mechanisms along with surface layers of a Mollisol after long-term fertilization in Northeast China. Journal of Soils and Sediments.

[ref-2] Adamczyk B, Heinonsalo J, Simon J (2020). Mechanisms of carbon sequestration in highly organic ecosystems—importance of chemical ecology. Chemistry Open.

[ref-3] An S, Mentler A, Mayer H, Blum WEH (2010). Soil aggregation, aggregate stability, organic carbon and nitrogen in different soil aggregate fractions under forest and shrub vegetation on the Loess Plateau, China. Catena.

[ref-4] Billings SA, Richter DD (2006). Changes in stable isotopic signatures of soil nitrogen and carbon during 40 years of forest development. Oecologia.

[ref-5] Breecker DO, Bergel S, Nadel M, Tremblay MM, Osuna-Orozco R, Larson TE, Sharp ZD (2015). Minor stable carbon isotope fractionation between respired carbon dioxide and bulk soil organic matter during laboratory incubation of topsoil. Biogeochemistry.

[ref-6] Čakmak D, Beloica J, Perović V, Kadović R, Mrvić VV, Knezević JB, Belanović S (2014). Atmospheric deposition effects on agricultural soil acidification state—key study: Krupanj Municipality. Achieves of Environmental Protection.

[ref-7] Calvin M, Benson AA (1948). The path of carbon in photosynthesis. Science.

[ref-8] Chang J, Zhu J, Xu L, Su H, Gao Y, Cai X, Peng T, Wen X, Zhang J, He N (2018). Rational land-use types in the karst regions of China: Insights from soil organic matter composition and stability. Catena.

[ref-9] Chang X, Chai Q, Wu G, Zhu Y, Li Z, Yang Y, Wang G (2017). Soil organic carbon accumulation in abandoned croplands on the loess plateau. Land Degradation & Development.

[ref-10] Chen H, Zhang W, Wang K, Hou Y (2012). Soil organic carbon and total nitrogen as affected by land use types in karst and non-karst areas of northwest Guangxi, China. Journal of the Science of Food and Agriculture.

[ref-11] Chen Q, Shen C, Peng S, Sun Y, Yi W, Za Li, Jiang M (2002). Soil organic matter turnover in the subtropical mountainous region of south China. Soil Science.

[ref-12] Deng L, Liu G-B, Shangguan Z-P (2014). Land-use conversion and changing soil carbon stocks in China’s ‘Grain-for-Green’ program: a synthesis. Global Change Biology.

[ref-13] Doetterl S, Stevens A, Six J, Merckx R, Van Oost K, Casanova Pinto M, Casanova-Katny A, Muñoz C, Boudin M, Zagal Venegas E, Boeckx P (2015). Soil carbon storage controlled by interactions between geochemistry and climate. Nature Geoscience.

[ref-14] Favis-Mortlock D, Boardman J (1995). Nonlinear responses of soil erosion to climate change: a modelling study on the UK South Downs. Catena.

[ref-15] Francisco CAL, Loss A, Brunetto G, Gonzatto R, Giacomini SJ, Aita C, Piccolo MDC, Torres JLR, Marchezan C, Scopel G, Vidal RF (2022). Carbon and nitrogen in particle-size fractions of organic matter of soils fertilised with surface and injected applications of pig slurry. Soil Research.

[ref-16] Fu J, Jiang D, Huang Y (2014a).

[ref-17] Fu J, Jiang D, Huang Y (2014b). 1 km grid population dataset of China (2005, 2010). Acta Geographica Sinica.

[ref-18] Geisseler D, Linquist BA, Lazicki PA (2017). Effect of fertilization on soil microorganisms in paddy rice systems—a meta-analysis. Soil Biology and Biochemistry.

[ref-19] Gupta Choudhury S, Srivastava S, Singh R, Chaudhari SK, Sharma DK, Singh SK, Sarkar D (2014). Tillage and residue management effects on soil aggregation, organic carbon dynamics and yield attribute in rice—wheat cropping system under reclaimed sodic soil. Soil and Tillage Research.

[ref-20] Hall J, Reynolds B, Aherne J, Hornung M (2001). The importance of selecting appropriate criteria for calculating acidity critical loads for terrestrial ecosystems using the simple mass balance equation. Water, Air, & Soil Pollution: Focus.

[ref-21] Han G, Li F, Tang Y (2015). Variations in soil organic carbon contents and isotopic compositions under different land uses in a typical karst area in Southwest China. Geochemical Journal.

[ref-22] Han G, Tang Y, Liu M, Van Zwieten L, Yang X, Yu C, Wang H, Song Z (2020). Carbon-nitrogen isotope coupling of soil organic matter in a karst region under land use change, Southwest China. Agriculture, Ecosystems & Environment.

[ref-23] Han R, Xu Z (2022). Spatial distribution and ecological risk assessment of heavy metals in karst soils from the Yinjiang County, Southwest China. PeerJ.

[ref-24] Han R, Zhang Q, Xu Z (2023). Tracing Fe cycle isotopically in soils based on different land uses: insight from a typical karst catchment, Southwest China. Science of the Total Environment.

[ref-25] He S, Liang Z, Han R, Wang Y, Liu G (2016). Soil carbon dynamics during grass restoration on abandoned sloping cropland in the hilly area of the Loess Plateau, China. Catena.

[ref-26] He YT, Zhang WJ, Xu MG, Tong XG, Sun FX, Wang JZ, Huang SM, Zhu P, He XH (2015). Long-term combined chemical and manure fertilizations increase soil organic carbon and total nitrogen in aggregate fractions at three typical cropland soils in China. Science of the Total Environment.

[ref-27] Heckman K, Welty-Bernard A, Rasmussen C, Schwartz E (2009). Geologic controls of soil carbon cycling and microbial dynamics in temperate conifer forests. Chemical Geology.

[ref-28] Henn MR, Gleixner G, Chapela IH (2002). Growth-dependent stable carbon isotope fractionation by basidiomycete fungi: delta(13)C pattern and physiological process. Applied and Environmental Microbiology.

[ref-29] Hu N, Lan J (2020). Impact of vegetation restoration on soil organic carbon stocks and aggregates in a karst rocky desertification area in Southwest China. Journal of Soils and Sediments.

[ref-30] Huang J, Hartemink AE (2020). Soil and environmental issues in sandy soils. Earth-Science Reviews.

[ref-31] Jiang Q, Zhou P, Liao C, Liu Y, Liu F (2020). Spatial pattern of soil erodibility factor (K) as affected by ecological restoration in a typical degraded watershed of central China. Science of the Total Environment.

[ref-32] Johnson HS, Hatch MD (1969). The C_4_-dicarboxylic acid pathway of photosynthesis. Identification of intermediates and products and quantitative evidence for the route of carbon flow. Biochemical Journal.

[ref-33] Jou RM, Macario KD, Pessenda LC, Pereira MG, Lorente FL, Pedrosa R, Silva Neto ECD, Fallon S, Muniz MC, Cardoso RP, Felizardo JPS, Anjos RMD (2021). The use of carbon isotopes (^13^C, ^14^C) in different soil types and vegetation coverage in a montane atlantic forest region, Southeast Brazil. Quaternary Geochronology.

[ref-34] Kiani F, Ghezelseflo A (2016). Evaluation of soil erodibility factor (k) for loess derived landforms of Kechik watershed in Golestan Province, North of Iran. Journal of Mountain Science.

[ref-35] Kohn MJ (2010). Carbon isotope compositions of terrestrial C_3_ plants as indicators of (paleo)ecology and (paleo)climate. Proceedings of the National Academy of Science.

[ref-36] Lehmann J, Kleber M (2015). The contentious nature of soil organic matter. Nature.

[ref-37] Li D, Wen L, Yang L, Luo P, Xiao K, Chen H, Zhang W, He X, Chen H, Wang K (2017). Dynamics of soil organic carbon and nitrogen following agricultural abandonment in a karst region. Journal of Geophysical Research: Biogeosciences.

[ref-38] Li X, Han G, Liu M, Liu J, Zhang Q, Qu R (2022). Potassium and its isotope behaviour during chemical weathering in a tropical catchment affected by evaporite dissolution. Geochimica Et Cosmochimica Acta.

[ref-39] Li Z-W, Zhang G-H, Geng R, Wang H, Zhang XC (2015). Land use impacts on soil detachment capacity by overland flow in the Loess Plateau, China. Catena.

[ref-40] Liang Y, Pan F, He X, Chen X, Su Y (2016). Effect of vegetation types on soil arbuscular mycorrhizal fungi and nitrogen-fixing bacterial communities in a karst region. Environmental Science and Pollution Research.

[ref-41] Liu M, Han G, Li X (2021). Comparative analysis of soil nutrients under different land-use types in the Mun River basin of Northeast Thailand. Journal of Soils and Sediments.

[ref-42] Liu M, Han G, Zhang Q (2020). Effects of agricultural abandonment on soil aggregation, soil organic carbon storage and stabilization: results from observation in a small karst catchment, Southwest China. Agriculture, Ecosystems & Environment.

[ref-43] Liu W, Xu Z, Jiang H, Zhou X, Zhao T, Li Y (2023). Lithological and glacial controls on sulfide weathering and the associated CO_2_ budgets in the Tibetan Plateau: new constraints from small catchments. Geochimica Et Cosmochimica Acta.

[ref-44] Liu W, Xu Z, Sun H, Zhao T, Shi C, Liu T (2018). Geochemistry of the dissolved loads during high-flow season of rivers in the southeastern coastal region of China: anthropogenic impact on chemical weathering and carbon sequestration. Biogeosciences.

[ref-45] Loss A, Lourenzi CR, Dos Santos E, Mergen CA, Benedet L, Pereira MG, Piccolo MDC, Brunetto G, Lovato PE, Comin JJ (2017). Carbon, nitrogen and natural abundance of ^13^C and ^15^N in biogenic and physicogenic aggregates in a soil with 10 years of pig manure application. Soil and Tillage Research.

[ref-46] Ni J, Luo DH, Xia J, Zhang ZH, Hu G (2015). Vegetation in karst terrain of southwestern China allocates more biomass to roots. Solid Earth.

[ref-47] Pal SC, Chakrabortty R (2019). Simulating the impact of climate change on soil erosion in sub-tropical monsoon dominated watershed based on RUSLE, SCS runoff and MIROC5 climatic model. Advances in Space Research.

[ref-48] Panagos P, Meusburger K, Ballabio C, Borrelli P, Alewell C (2014). Soil erodibility in Europe: a high-resolution dataset based on LUCAS. Science of the Total Environment.

[ref-49] Peri PL, Ladd B, Pepper DA, Bonser SP, Laffan SW, Amelung W (2012). Carbon (*δ*^13^C) and nitrogen (*δ*^15^N) stable isotope composition in plant and soil in Southern Patagonia’s native forests. Global Change Biology.

[ref-50] Philben M, Bowering K, Podrebarac FA, Laganière J, Edwards K, Ziegler SE (2022). Enrichment of ^13^C with depth in soil organic horizons is not explained by CO_2_ or DOC losses during decomposition. Geoderma.

[ref-51] Qu R, Han G (2022). Potassium isotopes in herbaceous plants: a potential new tool for C_3_ and C_4_ plant research. Journal of Geophysical Research: Biogeosciences.

[ref-52] Qu R, Han G (2023). Potassium isotopes of fertilizers as potential markers of anthropogenic input in ecosystems. Environmental Chemistry Letters.

[ref-53] Scharlemann JPW, Tanner EVJ, Hiederer R, Kapos V (2014). Global soil carbon: understanding and managing the largest terrestrial carbon pool. Carbon Management.

[ref-54] Schjønning P, Thomsen IK (2013). Shallow tillage effects on soil properties for temperate-region hard-setting soils. Soil and Tillage Research.

[ref-55] Sun L, Zhang G-H, Luan L-L, Liu F (2016). Temporal variation in soil resistance to flowing water erosion for soil incorporated with plant litters in the Loess Plateau of China. Catena.

[ref-56] Sun W, Zhu H, Guo S (2015). Soil organic carbon as a function of land use and topography on the Loess Plateau of China. Ecological Engineering.

[ref-57] Van Bavel CHM (1950). Mean weight-diameter of soil aggregates as a statistical index of aggregation. Soil Science Society of America Journal.

[ref-58] Wang B, Zhu Y, Erdenebileg E, Shi C, Shan D, Yang X (2022a). Effect of soil physicochemical properties on the steppe grazing potential in eastern Eurasian steppe. Journal of Soils and Sediments.

[ref-59] Wang G, Mao J, Fan L, Ma X, Li Y (2022b). Effects of climate and grazing on the soil organic carbon dynamics of the grasslands in Northern Xinjiang during the past twenty years. Global Ecology and Conservation.

[ref-60] Wang J, Cao L, Liu Y, Zhang Q, Ruan R, Luo X (2021). Effect of acclimatized paddy soil microorganisms using swine wastewater on degradation of rice straw. Bioresource Technology.

[ref-61] Wang Q-C, Wang W, Zheng Y, Vancov T, Fang Y, Xia Y, Liu X, Fan Y, Wei Z, Yang L (2022c). Converting rice paddy to upland fields decreased plant lignin but increased the contribution of microbial residue to SOC. Geoderma.

[ref-62] Wu L, Wang S, Bai X, Luo W, Tian Y, Zeng C, Luo G, He S (2017). Quantitative assessment of the impacts of climate change and human activities on runoff change in a typical karst watershed, SW China. Science of the Total Environment.

[ref-63] Wynn JG (2007). Carbon isotope fractionation during decomposition of organic matter in soils and paleosols: Implications for paleoecological interpretations of paleosols. Palaeogeography, Palaeoclimatology, Palaeoecology.

[ref-64] Xiao J, Zhao Y, Wang X, Hao Z, Wang K, Jiang S, Liu H, Zhou X (2022). Effects of recovery models on organic carbon pathways: a method using 13C natural abundance. Agriculture, Ecosystems & Environment.

[ref-65] Xu X, Shi Z, Li D, Rey A, Ruan H, Craine JM, Liang J, Zhou J, Luo Y (2016). Soil properties control decomposition of soil organic carbon: results from data-assimilation analysis. Geoderma.

[ref-66] Yakir D, Sternberg LDSL (2000). The use of stable isotopes to study ecosystem gas exchange. Oecologia.

[ref-67] Zeng J, Han G, Zhang S, Qu R (2023). Nitrate dynamics and source identification of rainwater in Beijing during rainy season: insight from dual isotopes and Bayesian model. Science of the Total Environment.

[ref-68] Zhang J, De Angelis D, Zhuang J, Zhang J, De Angelis D, Zhuang J (2011). Spatial variability of soil erodibility (K Factor) at a catchment scale in Nanjing, China. Theory and practice of soil loss control in Eastern China.

[ref-69] Zhang K, Shu A, Xu X, Yang Q, Yu B (2008). Soil erodibility and its estimation for agricultural soils in China. Journal of Arid Environments.

[ref-70] Zhang Z, Huang X, Zhou Y, Zhang J, Zhang X (2019). Discrepancies in Karst soil organic carbon in southwest china for different land use patterns: a case study of Guizhou province. International Journal of Environmental Research and Public Health.

[ref-71] Zhao Z, Zhao Z, Fu B, Wang J, Tang W (2021). Characteristics of soil organic carbon fractions under different land use patterns in a tropical area. Journal of Soils and Sediments.

